# Selected osteointegration markers in different timeframes after dental implantation: findings and prognostic value

**DOI:** 10.3389/abp.2024.12433

**Published:** 2024-02-05

**Authors:** Emir Bayandurov, Zurab Orjonikidze, Sophio Kraveishvili, Ramaz Orjonikidze, George Ormotsadze, Sophio Kalmakhelidze, Tamar Sanikidze

**Affiliations:** ^1^ Department of Oral Surgery and Implantology, Tbilisi State Medical University, Tbilisi, Georgia; ^2^ Department of Physics, Biophysics, Biomechanics and Informational Technologies, Tbilisi State Medical University, Tbilisi, Georgia

**Keywords:** osteopontin, osteocalcin, bone alkaline phosphatase, osteoprotegerin, nitric oxide

## Abstract

The study aimed to determine the osteointegration markers after dental implantation and evaluate their predictive value. The study was performed on 60 practically healthy persons who needed teeth rehabilitation using dental implants. The conical-shaped implants (CI) and hexagonal implants (HI) were used. The content of Osteopontin (OPN), Osteocalcin (OC), Alkaline Phosphatase (ALP), Osteoprotegerin (OPG), and nitric oxide (NO) was determined in patients’ gingival crevicular fluid (GCF) and peri-implant sulcular fluid (PISF), collected 1, 3, and 6 months after implantation. During the 3–6 months of observation level of OPN increased in patients with CIs (<50 years > 50 years) and HIs (<50 years) (CI: <50 years F = 36.457, *p* < 0.001; >50 years F = 30.104, *p* < 0.001; HI < 50 years F = 2.246, *p* < 0.001), ALP increased in patients with CIs (<50 years: F = 19.58, *p* < 0.001; >50 years: F = 12.01; *p* = 0.001) and HIs (<50 years) (F = 18.51, *p* < 0.001), OC increased in patients <50 years (CI: F = 33.72, *p* < 0.001; HI: F = 55.57, *p* < 0.001), but in patients >50 years - on the 3 days month (CI: F = 18.82, *p* < 0.001; HI: F = 26.26, *p* < 0.001), but sharply decreased at the end of sixth month. OPG increased during 1–3 months of the observation in patients <50 years (CI: F = 4.63, *p* = 0.037; HI: F = 2.8927, *p* = 0.046), but at the end of the sixth month returned to the initial level; NO content in PISF increased in patients with CI (>50 years) during 1–6 months of the observation (F = 27.657, *p* < 0.001). During the post-implantation period, age-related differences in osteointegration were observed. Patients <50 years old had relatively high levels of OPN, ALP, OC, and OPG in PISF, resulting in less alveolar bone destruction around dental implants and more intensive osteointegration. These indicators may be used as biological markers for monitoring implant healing. The process of osseointegration was more intense in CIs due to their comparatively high mechanical loading.

## Introduction

Aesthetic and functional rehabilitation using dental implants offers highly predictable and esthetical results; hence it has become one of the alternatives to be included in the therapeutic options for the treatment of totally or partially edentulous patients. Despite this, the risk of failure remains high but difficult to predict (Scarano et al., 2023).

The implant’s stability significantly depends on the osteointegration process between the bone and the implant. Osteointegration is a continuous process of osteoclast and osteoblast activation, necessary for bone repair, formation, and functional recovery (Martin and Sims, 2005).

Bone remodeling is critical to maintaining long-term stable osseointegration. The mechanisms of wound healing around dental implants are characterized by several features. The cellular and molecular mechanisms of the osseointegration process have not yet been fully established and require further research in this direction (Matsuura and Yamashita, 2018).

Early monitoring of sensitive clinical-laboratory indicators of patients with dental implants, correlated with pathological disorders in the osteointegration process in the early stages of the implantation, can be used to identify predictive markers of possible complications and their severity at its later stages. This can be useful for providing guides to treatment strategies and preventing complications, maintaining long-term stable osseointegration.

Since the array of clinical indices of periodontal origin, such as indices recording gingival inflammation, plaque accumulation, and bleeding and probing depths (clinical index) most frequently gives the possibility only determining the soft tissue inflammatory response, rather than detecting its early predictors (Greenstein, 1996), for the determination of peri-implant clinical status, various oral fluids are used, the molecules of which are associated with the inflammatory response, bone metabolism, and proteinases.

The gingival crevicular fluid is the osmotically mediated physiological exudate originating from serum and tissue fluid that seeps through the crevicular and junctional epithelium. Gingival crevicular fluid plays a special part in maintaining the structure of junctional epithelium and the antimicrobial defense of periodontium, reflects the cellular response in the periodontium by the constituents from the gingival crevice, and is an important determinant of the status of periodontal tissues (Akman et al., 2018). The peri-implant sulcus is, anatomically, functionally, and environmentally quite similar to periodontal crevices (Berglundh et al., 1991; Piattelli et al., 1996; Buser et al., 1997); this fluid was termed the peri-implant sulcular fluid. Peri-implant sulcular fluid like gingival crevicular fluid is composed of serum and locally generated materials such as tissue breakdown products, inflammatory mediators (cytokines, prostaglandins), tissue degradation components, mineralized tissue components, bone turnover markers, and antibodies directed against dental plaque bacteria (Akman et al., 2018; Subbarao et al., 2019). Peri-implant sulcular fluid analysis can potentially reflect the actual status of peri-implant soft and hard tissues (Lang and Berglundh, 2011). GCF and PISF could be useful markers of early inflammation in gingival and peri-implant tissues (Shama et al., 2016).

The study aimed to determine the osteointegration markers in different timeframes after dental implantation and their prognostic value.

## Materials and methods

The study was performed on 60 persons (aged from 18 to 65 years) who performed teeth rehabilitation using dental implants based on Dental Clinic and Training-Research Center UniDent and Dental Clinic A1 during 2020–2022 years.

### Patient inclusion criteria in the study

Practically healthy male persons (without comorbidities) (60 patients) who needed rehabilitation using dental implants in the chewing teeth in the mandible, with good hygiene of the oral cavity (Board index <20%) (Ainamo and Bay, 1975) were included in the study.

### Patients excluding criteria in the study

Females were excluded from the study to avoid the possible influence of pre-menopause/menopause-related hormonal imbalances on the process of osseointegration. Patients with various accompanying diseases (allergy, cancer, hepatitis, diabetes, endocrine system disorders, stomach ulcer, chronic gastritis, colitis, respiratory diseases, and pregnant women), also were patients who, during the last 6 months before implantation, used medications that can change the osseointegration (including anti-inflammatory drugs) were excluded from the study.

The research plan was approved by the Ethics Committee of Tbilisi State Medical University. All subjects signed informed consent.

The conical-shaped implants (CIs) (<50 years—10 patients, >50 years—20 patients) and hexagonal implants (HIs) (<50 years—15 patients, >50 years—15 patients) from the AlphaBio were used.

The selected age range (18–65 years) lowered chronic comorbidity risk. The age limit of 50 years was chosen to equally distribute patients between groups while considering the mean number of patients of different ages. This distribution of the patients will give the possibility to assess the dynamic intensity of osteointegration processes with aging.

The GSF and PSF were collected from patients 1, 3, and 6 months after implantation, and the content of Osteopontin (OPN), Osteocalcin (OC), bone Alkaline Phosphatase (ALP), Osteoprotegerin (OPG), and nitric oxide (NO) was determined.

### Collection of gingival crevicular fluid (GCF) and peri-implant sulcular fluid (PISF)

PISF and GCF were obtained by the method considering minimal mechanical irritation (Rudin et al., 1970). The area to be sampled was treated with sterile cotton swabs to remove dental plaque and then air-dried to prevent plaque and saliva contamination. To obtain a sample PISF strips of standardized paper (Periopaper, no. 593525) were placed at the entrance of the grooves of the implant and healthy teeth and inserted to a standardized depth of 1 mm at each site regardless of probing depth to avoid further mechanical irritation. To obtain a sample GCF strips of standardized paper (Periopaper, no. 593525) were placed at the entrance of the grooves between the healthy teeth and inserted to a standardized depth of 1 mm at each site egardless of probing depth to avoid further mechanical irritation. The sampling time is standardized and equal to 30 s. Samples contaminated with blood were not used.

For safe storage of PISF and GCF samples, the paper strips will be placed in sterile Eppendorf and stored at −80°C until laboratory analysis.

### Analysis

After the paper strips in Eppendorf tubes were kept at room temperature for at least 30 min, 100 μL assay buffer included in the kit was added to each Eppendorf tube and put into the shaker device for 45 min. Then the tubes were centrifuged at 11,200 rpm for 15 min. After the GCF/PISF in the paper strips were transferred to the assay buffer, the assay buffer in the Eppendorf was taken using a clean polypropylene pipette, and the levels of OPN, OC, bone ALP, and OPG were measured according to the manufacturer’s instructions.

The bone markers in PISF and GCF samples were determined by immuno-enzymatic method (ELISA) using HumaReader SETPROD immune-enzymatic counter. OPG was determined by use of EH0247 Human OPG ELISA Kit OC—EH3468 Human OC/BGP ELISA Kit, OPN—EH0248 Human OPN ELISA Kit, bone ALP—BIOLABO Kit.

For the determination of NO content in PISF and GCF, distilled water (130 µL per sample) was added to the Eppendorfs containing PISF and GCF, and the Eppendorfs were shaken vigorously to dissolve the nitrite in the water. To the 100 µL of the obtained extract, 0.5 mL of freshly prepared Greiss reagent was added; after a 10-minute incubation at room temperature, the absorbance intensity of each sample placed on the microplate was measured at a wavelength of 540 nm (Grisham et al., 1996). A standard curve was prepared using sodium nitrite to calculate the nitrite (NO_x_) concentration in the GSF and PISF.

### Statistical analysis

Statistical significance was tested using analysis of variance ANOVA and a two-sample *t*-test. Relationships yielding *p*-values less than 0.05 were considered significant. All values were expressed as the mean ± SE.

## Results

As follows from the data presented in Table 1 the content of OPN, OC, bone ALP, OPG, and NO in the gingival crevicular fluid did not change statistically significantly during the entire observation period.

**TABLE 1:** Statistical significance of OC, OPG, OPN, ALP, and NO in the gingival crevicular fluid alterations after the 6 months after implantation (analysis of variance (ANOVA) F, between-group variability/within-group variability; *p*, level of significance of Null Hypothesis).

VAR	AGE <50		Age ≥ 50	
	*F*	*p*	*F*	*p*
OC	0.059	0.942	0.366	0.698
OPG	0.022	0.977	0.027	0.972
OPN	0.154	0.858	0.036	0.964
ALP	0.010	0.989	0.130	0.878
NO	0.884	0.438	0.134	0.875

**FIGURE 1:**
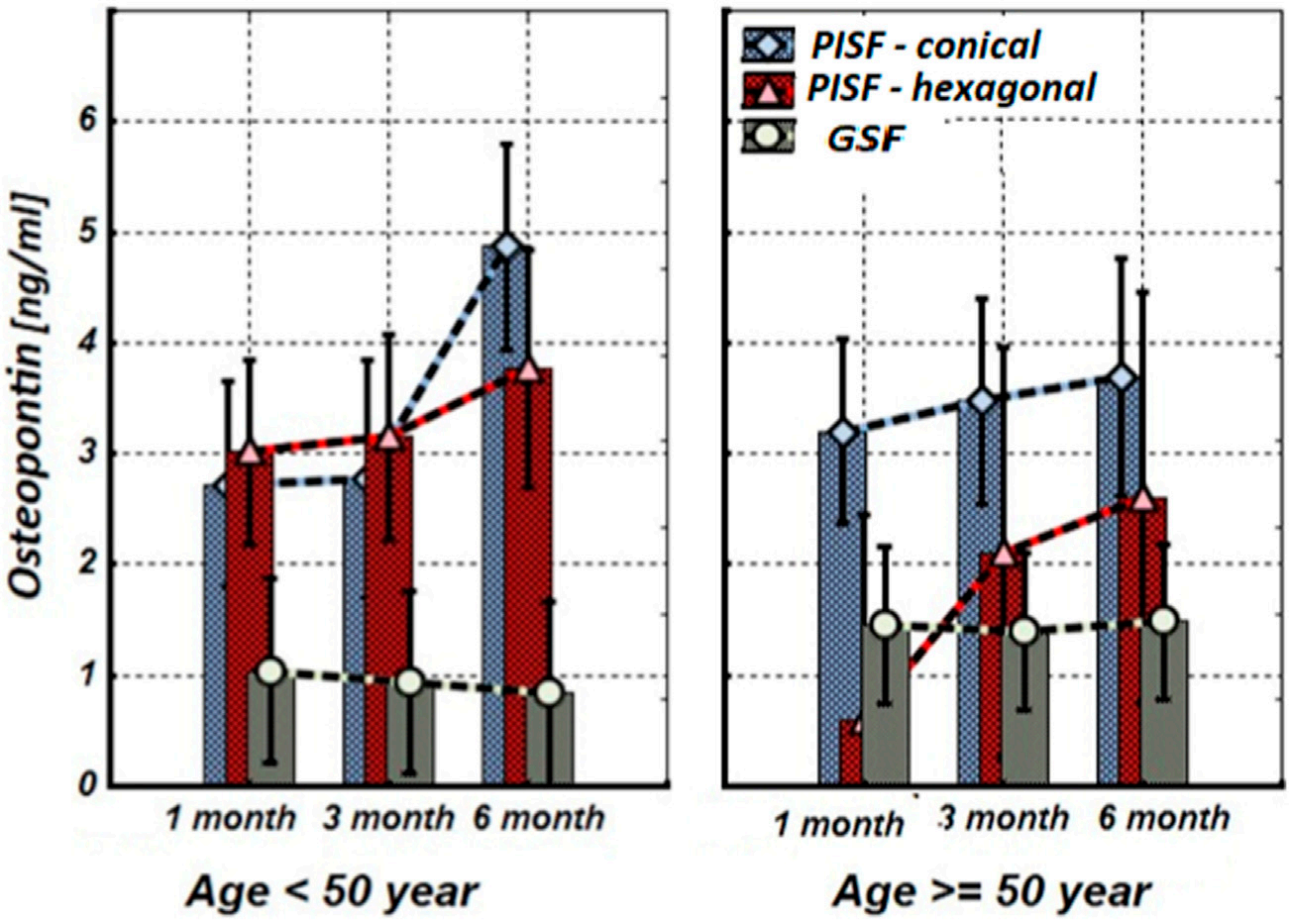
The levels of OPN in the gingival crevicular fluid (GCF) and peri-implant sulcular fluid (PISF) of patients of different age groups during the post-implantation period (after 1, 3, and 6 months), in cases of conical-shaped implants (CIs) and hexagonal implants (HIs).

**FIGURE 2:**
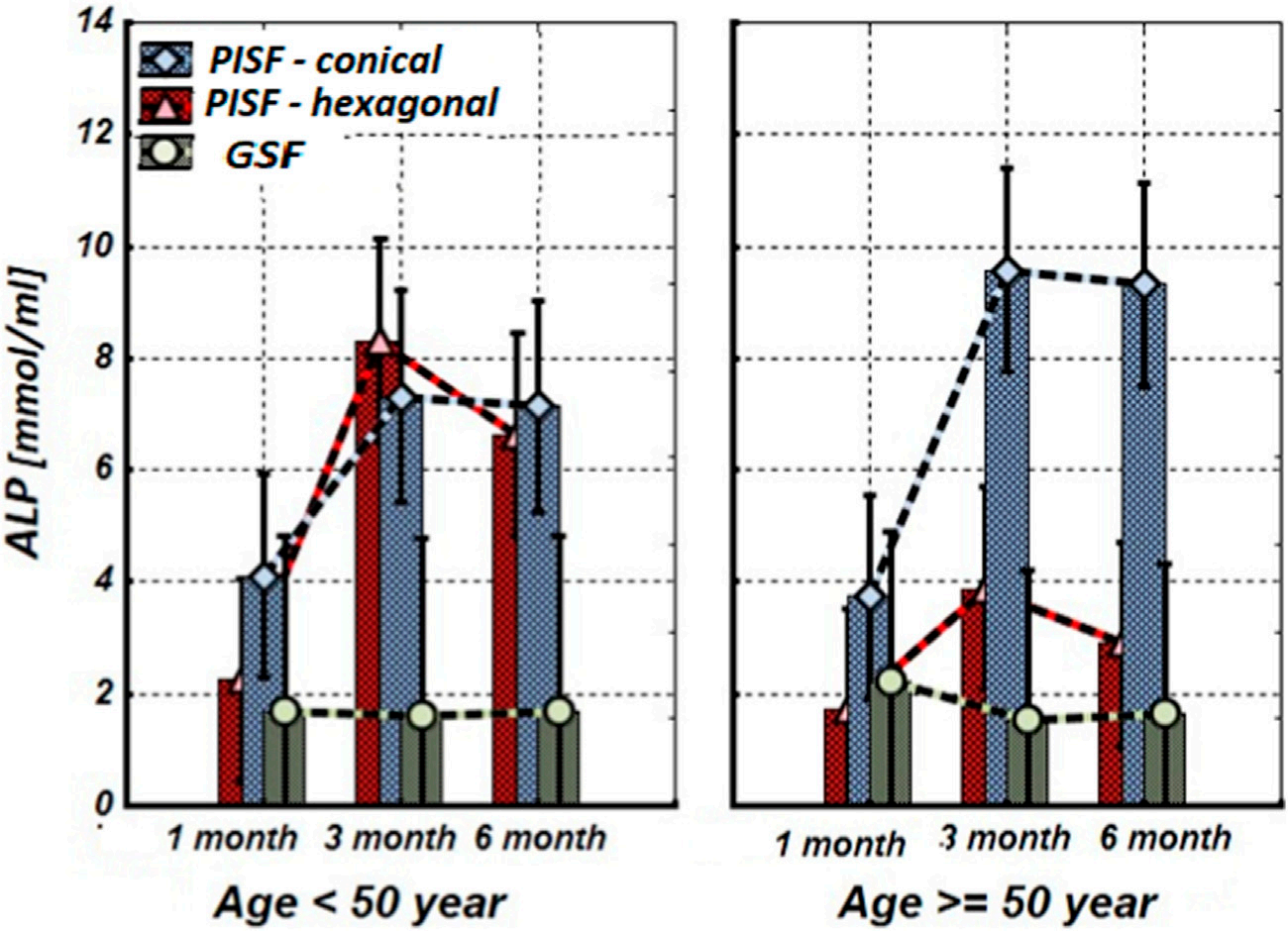
The levels of ALP in the gingival crevicular fluid (GCF) and peri-implant sulcular fluid (PISF) of patients of different age groups during the post-implantation period (after 1, 3, and 6 months), in cases of conical-shaped implants (CIs) and hexagonal implants (HIs).

**FIGURE 3:**
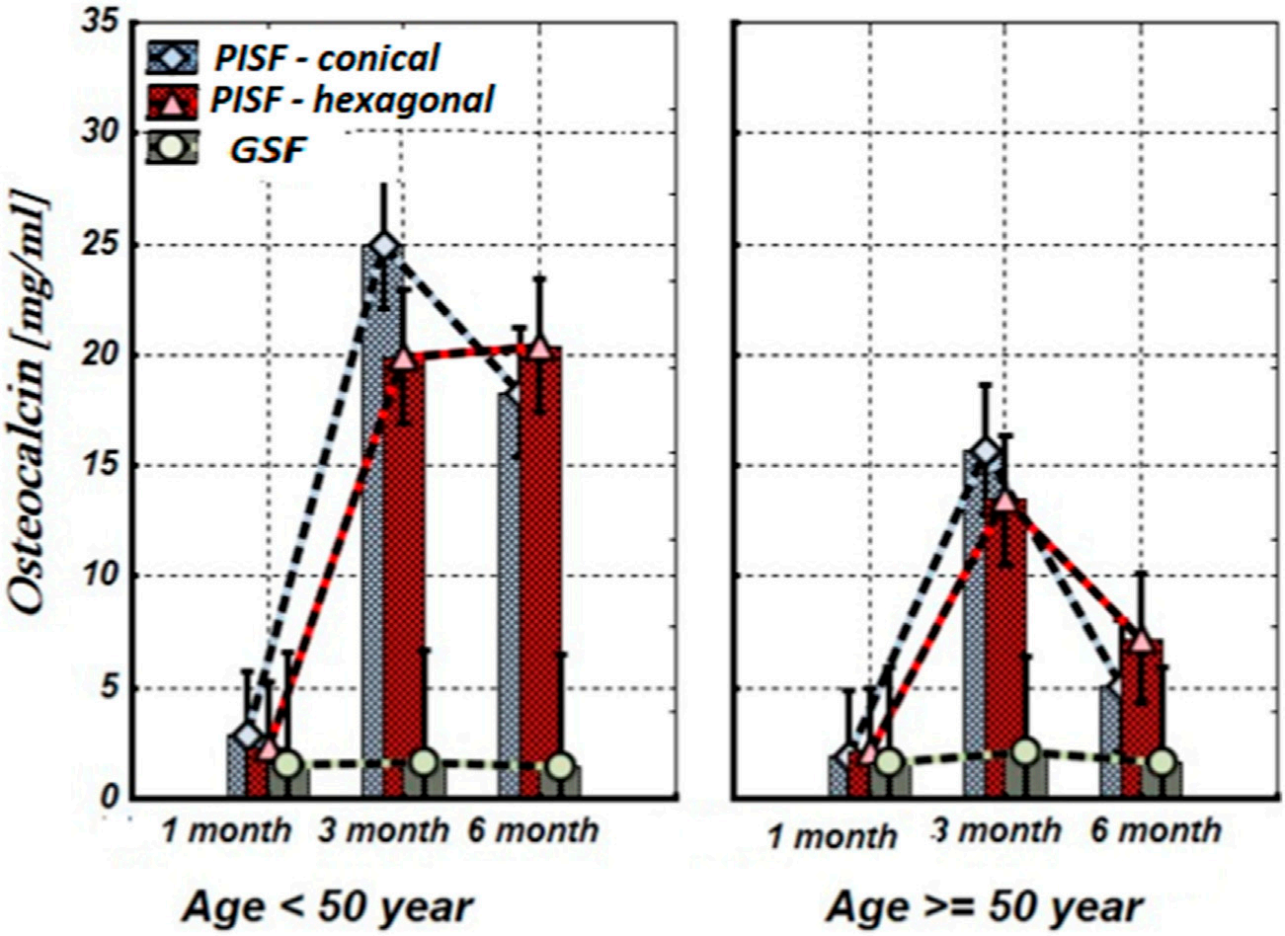
The levels of OC in the gingival crevicular fluid (GCF) and peri-implant sulcular fluid (PISF) of patients of different age groups during the post-implantation period (after 1, 3, and 6 months), in cases of conical-shaped implants (CIs) and hexagonal implants (HIs).

**FIGURE 4:**
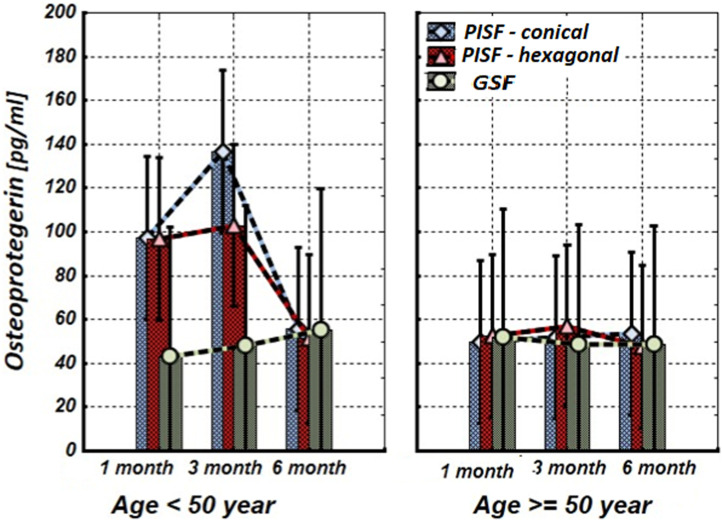
The levels of OPG in the gingival crevicular fluid (GCF) and peri-implant sulcular fluid (PISF) of patients of different age groups during the post-implantation period (after 1, 3, and 6 months), in cases of conical-shaped implants (CIs) and hexagonal implants (HIs).

**FIGURE 5:**
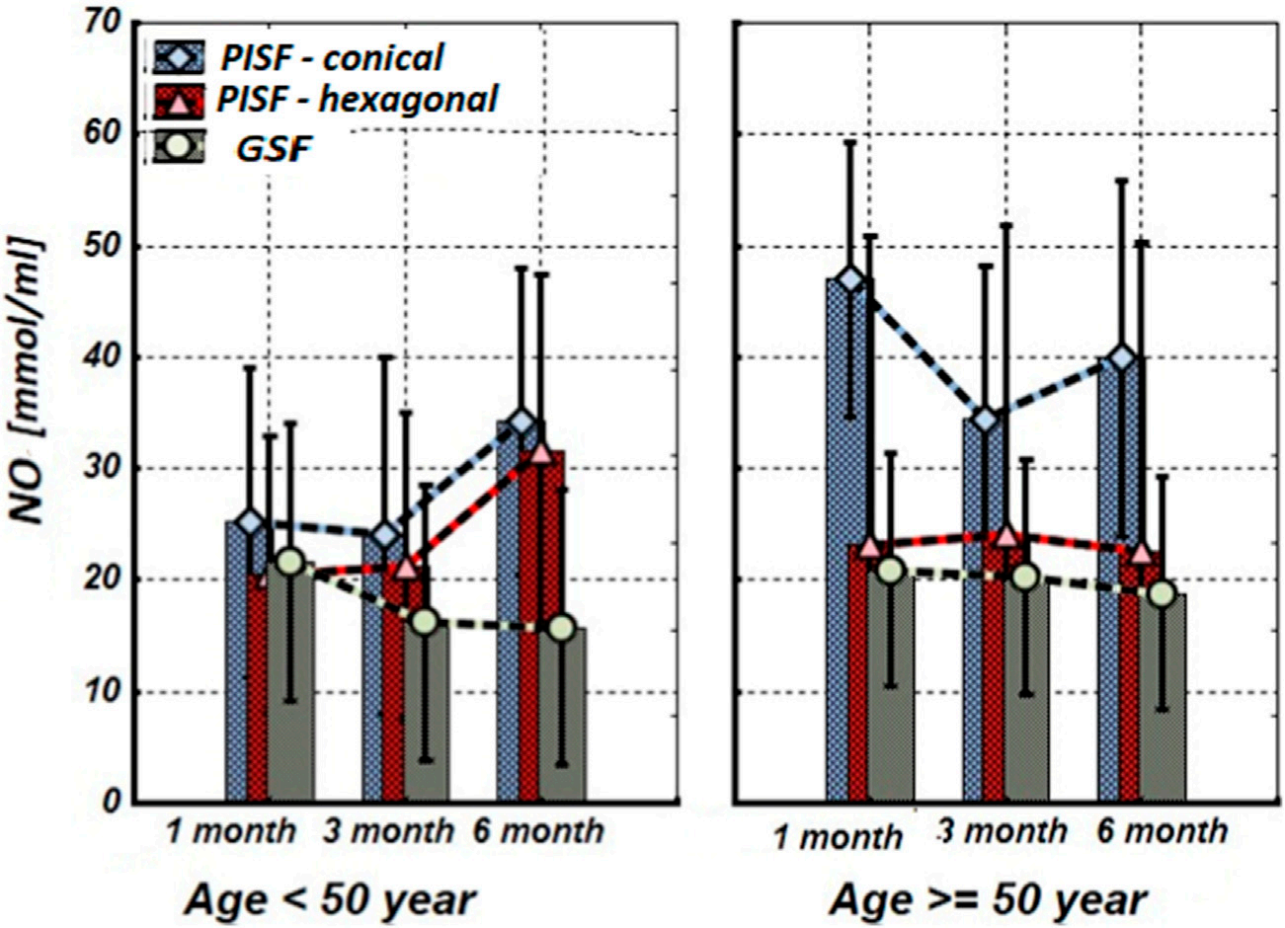
The levels of nitrite (NO_x_) concentration in the gingival crevicular fluid (GCF) and peri-implant sulcular fluid (PISF) of patients of different age groups during the post-implantation period (after 1, 3, and 6 months), in cases of conical-shaped implants (CIs) and hexagonal implants (HIs).

Figures 1–5 show alterations of OPN, OC, bone ALP, and OPG, and NO content in the patients’ GSF and PISF after 1, 3, and 6 months after implantation.

Figure 1 shows that after the implantation the level of OPN in patients’ peri-implant sulcular fluid (PISF) increased in comparison to its level in gingival crevicular fluid (GSF). In the case of the CIs in both age groups the content of OPN in PISF increased during the entire observation period (1–6 months) and reached a maximum by the end of 6 months of observation (<50 years—F = 36.457; *p* < 0.001; >50 years—F = 30.104; *p* < 0.001), but in case of HIs, an increase in the OPN content was recorded during the observation period only in patients of the age group <50 years (F = 22.246; *p* < 0.001); in age group >50 years, an increase in the level of OPN in PISF was not statistically significant.

Figure 2 shows that during the first month after implantation, the content of ALP in peri-implant sulcular fluid (PISF) did not change significantly in comparison to its level in gingival crevicular fluid (GSF) (CI: <50 years—F = 1.15; *p* = 0.297; >50 years—F = 1.35; *p* = 0.258; HI: <50 years—F = 2.43; *p* = 0.135; >50 years—F = 2.99; *p* = 0.099) and increased in both age groups at 3 months after the implantation. In patients with CIs, this tendency continued for 3–6 months and was statistically significant in both age groups (<50 years—F = 19.58; *p* < 0.001; >50 years—F = 12.01; *p* = 0.001). In patients with HIs during the 3–6-month period in the age group <50 years the ALP content in PISF increased significantly (F = 18.51; *p* < 0.001), but in patients older than 50 years the statistical significance of the difference was not high (F = 1.30; *p* = 0.233).

As follows from Figure 3, 1 month after implantation OC levels in peri-implant sulcular fluid (PISF) did not differ from the corresponding values in gingival crevicular fluid (GCF) (conical: F = 2.065; *p* = 0.164; hexagonal: F = 1.107; *p* = 0.304); its content began to increase only 3–6 months after the implantation. In patients under the age of 50 years the level of OC in PISF was high during the 3–6 months of the observation period (CI: F = 33.72; *p* < 0.001; HI: F = 55.57; *p* < 0.001), whereas in patients of the age group after 50 years, the level of OC in PISF increased up to the 3 months of observation (conical: F = 18.82; *p* < 0.001; hexagonal: F = 26.26; *p* < 0.001), and sharply decreased by the end of 6 months (CI: F = 1.10; *p* = 0.261; HI: F = 1.71; *p* = 0.204).

As follows from Figure 4, during the first 3 months of observation (1–3 months) OPG in peri-implant sulcular fluid (PISF) in patients of the age group <50 years increased (CI: F = 4.63; *p* = 0.037; HI: F = 2.8927; *p* = 0.046) compared to its level in gingival crevicular fluid (GCF), but by the end of the sixth month it was returned to the initial level (CI: F = 1.03; *p* = 0.29; HI: F = 1.07; *p* = 0.31). In patients of the age group >50, the level of OPG in PISF did not change statistically significantly compared to its level in GCF during the entire follow-up period (CI: F = 1.23; *p* = 0.246; HI: F = 1.03; *p* = 0.301).

As follows from Figure 5, in patients of the age group <50 years, the mean values of the nitrite (NO_x_) concentration in peri-implant sulcular fluid (PISF) did not changed during whole observation period compared with the corresponding values in gingival crevicular fluid (GCF) only at the end (6-th months) after implantation (CI: F = 1.615; *p* = 0.2443; HI: F = 1.524; *p* = 0.263). In patients of the age group >50 years with a CIs, the NO_x_ concentration in PISF increased statistically significantly compared to the corresponding values in GCF during the whole period (1–6 months) of the observation (F = 27.657; *p* < 0.001), while in the case of a HIs, did not statistically significantly change compared to the corresponding values in GCF (F = 0.596; *p* = 0.448).

## Discussion

The success of the osteogenic process requires careful chronological coordination of molecular signals to drive the proliferation, migration, and differentiation of mesenchymal precursor cells in osteoblasts (Zaidi, 2007). In the last decades, periodontal research has focused on the analysis of potential host markers that can be used to diagnose the healing intensity of implants and determine prognosis (Arikan et al., 2008).

Bone is in a constant state of remodeling, which is important for the maintenance of its normal structure and function. Many types of cells and factors are involved in the process of bone remodeling.

Osteoblasts, responsible for new bone formation, and osteoclasts responsible for bone resorption, are the two main cell types participating in those processes (Matsuoka et al., 2014; Chen et al., 2018). These processes are stable and balanced under physiological conditions; However, bone architecture or function will be disturbed when the balance is disordered.

The process of osseointegration begins after the placement of the dental implant in the jaw bone; the implant integrates with the living bone by healing the bone wound. OPN and OC are major non-collagenous proteins involved in bone matrix organization and deposition. They are produced during bone formation, later in the mineralization process, and involved in organizing the extracellular matrix and coordinating cell-matrix, mineral–matrix interactions, which play key roles in the biological and mechanical functions of bone, regulate whole-bone structure and morphology (Bailey et al., 2017).

At first, OPN is secreted on the hard surface of the bone. This extracellular matrix protein is a major factor affecting osteoclast attachment, wound healing, and angiogenesis, plays an important role in bone mineralization, cell adhesion, differentiation, and foreign body response, since it has several binding sites with implant hydroxyapatite crystals, collagen, and various integrins, through the calcium ions and Arg-Gly-Asp motif (Makishi et al., 2022).

Secretion of OPN at the resorption site during bone remodeling regulates migration, adhesion, differentiation, and activation of osteoclasts to form a bone matrix, also, migration, adhesion, and differentiation of osteoblasts. OPN positively affects osteoblasts in direct osteogenesis after implantation (Dodds et al., 1995; McKee and Nanci, 1995).

As follows from the results of our study level of OPN in patients’ PISF increased in comparison to its level in GSF from the early stages after the implantation. In the case of CIs, the content of OPN in PISF was higher compared to the corresponding values in GCF during the entire observation period (1–6 months) and reached a maximum by the end of sixth month at both age groups, but in the case of HIs, an increase in the OPN content in PISF from the beginning to the end of the observation (sixth month), was recorded only in patients of the age group <50 years, in age group >50 years, an increase in the level of OPN in PISF was not statistically significant.

During bone mineralization, osteoblasts secrete a specific membrane-bound glycoprotein, ALP, that catalyzes the hydrolysis of phosphate monoesters and supports a high concentration of phosphate on the surface of osteoblasts (Sharma et al., 2014). Elevated levels of ALP in sulcular fluid are observed as a compensatory mechanism in response to destructive disease processes and indicate active bone formation (Malik et al., 2015).

According to our study results, OC and ALP content increase in PISF began 3 months after implantation. In patients with CIs, ALP remains this statistically significant tendency for 3–6 months in both age groups, however, in patients with HIs in patients older than 50 years the statistical significance of this growth was not high. Regarding OC, under the age of 50 years in patients with conical-shaped and hexagonal implants, the level of OC in PISF was high during 3–6 months after implantation, whereas in patients of the age group after 50 years, the level of OC in PISF increased on to the 3 months of observation, and sharply decreased at the end of 6 months. In literature, age-related decline of OC has been established (Vanderschueren et al., 1990). A weak correlation between the bone markers ALP and OC levels, and the dental implant stability quotient (ISQ) over the healing period was revealed, which is in harmony with the intensification of a gene expression of bone markers in PICF (Tirachaimongkol et al., 2016) and indicates that these biological markers may be used for monitoring of implant healing.

These data indicate that when using CIs, the process of osseointegration proceeds intensively in both age groups, while when using HIs, the intensity of this process in elderly patients decreases. This is due to the comparatively higher mechanical loading produced by the CIs (Bayandurov et al., 2023), as well as age-related characteristics of osteogenesis—in yang (<50 years) patients content of the OPG, ALP, and OC in PISF was especially high.

Osteointegration success is determined by the incorporation of the woven bone and the bone mass adaptation to bearing a load (Parithimarkalaignan and Padmanabhan, 2013). Disruption of the balance of factors regulating host response can induce impairment osteointegration process and bone remodeling, stimulation of osteoclasts activity with consequent alveolar bone resorption, and implant loss (Giannopoulou et al., 2012).

Osteoclastogenesis is coordinated by the interaction of three members of the tumor necrosis factor (TNF) superfamily: receptor activator of nuclear factor-kB (NF-kB) ligand (RANKL), RANK, and OPG. The RANK, RANKL, and OPG, known together as the RANK-RANKL-OPG system, effectively control the balance between osteoblasts and osteoclasts activity. RANKL is expressed by osteoblasts, stromal cells, fibroblasts, B cells, and T cells when stimulated by cytokines and bacterial lipopolysaccharides. The binding of RANK and RANKL on the surface of preosteoclast/osteoblast cells, activates the formation, maturation, and activation of osteoclasts, resulting in bone destruction. Conversely, OPG, produced by periodontal ligament cells, gingival fibroblasts, and epithelial cells, is a soluble circulating decoy receptor of RANK, blocks the activation of RANK by preferentially binding itself to RANKL, and thus protects bone against destruction. Therefore, RANKL and OPG regulate bone resorption by positive or negative stimulation of RANK on osteoclast cells (Giannopoulou et al., 2012). OPG is involved in the regulation of alveolar bone destruction around dental implants through the regulation of osteoclast differentiation.

As follows from the results of our study, in patients of the age group <50 years OPG in PISF during the first 3 months of observation (1–3 months) increased compared to its level in GCF, but by the end of the sixth month returned to the initial level. In patients of the age group >50, the level of OPG in PISF did not statistically significantly change compared to its level in GCF during the entire follow-up period. Consequently, in the early stages of osteointegration in young patients, OPG protects alveolar bone destruction around dental implants and promotes osteointegration, while in older patients the effectiveness of this mechanism decreases.

In the regulation of inflammation in soft tissues around the implant, immunomodulation, antimicrobial defense, as well as anabolic reactions, and bone resorption process important role plays NO, a small-sized, highly reactive molecule, that is a secondary messenger in a living organism. During implantation, it is possible an increase in NO production as a result of the intensification of iNOS activity induced by proinflammatory cytokines, or a decrease in NO content related to its conversion into peroxynitrite in inflammation-induced oxidative stress conditions (Allaker et al., 2001). Mechanical stimulus, one of the factors involved in the bone remodeling process around implants, mediates osteoclast activity and also stimulates NO production (Baloul, 2016; Gokmenoglu et al., 2018). Therefore, NO metabolism is associated with the clinical state of peri-implant tissues, NO seems to have a biphasic effect on osteoblastic activity. *In vitro*, studies have shown that a small amount of NO constitutively produced by osteoblasts, or slowly released by donors, can act as a stimulator of osteoblast growth and differentiation, while high NO concentration has a potent inhibitory effect on osteoblastic growth and differentiation, and/or stimulates bone resorption, that may be partly due to its pro-apoptotic effect. In inflamed peri-implant tissues, the NO level was found to be higher than in healthy sites (Nascimento et al., 2020).

Our study results show that in patients with CIs and HIs of the age group <50 years, the mean values of NO_x_ concentration in PISF statistically insignificantly increased compared with the corresponding values in GCF on the sixth month of the implantation. In patients of the age group >50 years CIs induced a statistically significant increase of NO_x_ concentration in PISF, while in the case of HIs, it did not change statistically significantly compared to the corresponding values in GCF during the entire observation period.

It can be assumed that in patients <50 years there is a slight increase in NO_x_ concentration in PISF stimulates osteoblast growth and differentiation, and therefore, the osteointegration process, whereas the statistically significant increase of NO_x_ concentration in PISF in >50 years old patients in the case of a CIs, may be related with the inflammatory reaction (which needs correction) or other factors (oxidative stress, mechanical stimulus, etc.) (Orjonikidze et al., 2020).

## Conclusion

During the post-implantation period, the process of osteointegration is affected by age-related factors. Patients <50 years have higher levels of OPN, ALP, OC, and OPG, which result in lesser alveolar bone destruction around dental implants and a more intensive osteointegration process. Biological markers such as OPN, OC, ALP, PNG, and NO content in PISF can be used to monitor implant healing. The use of CIs leads to a more intensive process of osseointegration due to the higher mechanical loading produced by them.

## Data Availability

The raw data supporting the conclusion of this article will be made available by the authors, without undue reservation.
